# Prediction of Protection against Asian Enterovirus 71 Outbreak Strains by Cross-neutralizing Capacity of Serum from Dutch Donors, The Netherlands

**DOI:** 10.3201/eid2209.151579

**Published:** 2016-09

**Authors:** Sabine M.G. van der Sanden, Gerrit Koen, Hetty van Eijk, Sylvie M. Koekkoek, Menno D. de Jong, Katja C. Wolthers

**Affiliations:** Academic Medical Center, Amsterdam, the Netherlands

**Keywords:** Picornaviruses, human enterovirus 71, seroprevalence, intravenous immunoglobulins, cross protection, cross-neutralization, Asian enterovirus 71, Dutch, the Netherlands, Europe, viruses, enterovirus

## Abstract

Outbreaks of human enterovirus 71 (EV-71) in Asia are related to high illness and death rates among children. To gain insight into the potential threat for the population of Europe, we determined the neutralizing activity in intravenous immunoglobulin (IVIg) batches and individual serum samples from donors in the Netherlands against EV-71 strains isolated in Europe and in Asia. All IVIg batches and 41%, 79%, and 65% of serum samples from children ≤5 years of age, women of childbearing age, and HIV-positive men, respectively, showed high neutralizing activity against a Dutch C1 strain, confirming widespread circulation of EV-71 in the Netherlands. Asian B3–4 and C4 strains were efficiently cross-neutralized, predicting possible protection against extensive circulation and associated outbreaks of those types in Europe. However, C2 and C5 strains that had few mutations in the capsid region consistently escaped neutralization, emphasizing the importance of monitoring antigenic diversity among circulating EV-71 strains.

## Abstract 2

Herd immunity induced by locally circulating strains could provide protection against introduction of new strains.

Enterovirus 71 (EV-71) is a member of the genus *Enterovirus*, family *Picornaviridae*, and is a major causative agent of hand, foot and mouth disease in children <5 years of age. The virus may also invade the central nervous system and cause severe neurologic disease, including paralysis and brainstem encephalitis (*1*). On the basis of nucleotide sequence diversity in the viral protein (VP)1 capsid gene, 7 EV-71 genogroups (A–G) have been defined (*2*,*3*). Genogroup A contains a prototype strain that was isolated in the United States in 1969; additional strains of this genogroup were identified in China in May 2008 (*4*). Genogroup B and C viruses have been circulating more widely and contain 6 and 5 genotypes, respectively, defined as B0–B5 and C1–C5. Within genotype C4, 2 additional subgenotypes have been classified, C4a and C4b (*2*). The genogroups D and G in India and genogroups E and F in Africa were identified more recently (*2*,*3*).

The incidence of EV-71 infection has greatly increased in the Asia–Pacific region since 1997. Multiple countries within this region have documented massive outbreaks of EV-71, reporting thousands of cases of severe illness and death among children (*1*). The increased incidence coincided with the identification of new genotypes (B3–5, C3–5) (*5*). Although strains of genotypes C4 and B5 have been isolated from patient samples in Europe, widespread circulation of new genotypes and associated massive outbreaks are restricted to the Asian Pacific region (*5*–*10*). Most EV-71 strains circulating in Europe belong to genotypes C1 and C2, and presence of herd immunity conferred by cross-protective antibodies induced by these types could explain the limited spread of new genotypes. However, this hypothesis has not been formally studied. Multiple studies have reported cross-neutralization, but antigenic diversity among different EV-71 genotypes has also been observed (*11*–*17*). These studies have mostly been conducted by using serum samples from Asian donors or animals immunized with Asian outbreak strains.

Intravenous administration of human immunoglobulin (IVIg) is currently the only option to treat persons with severe enterovirus infections. For that, the determination of the neutralizing capacity of IVIg batches against locally circulating strains is of clinical importance. Furthermore, IVIg used in the Netherlands contains plasma from >1,000 healthy Dutch donors and so represents the immunologic profile of the general population against specific pathogens. To gain more insight into the potential threat of Asian EV-71 outbreak strains for the European population and the potential treatment efficacy of IVIg, we determined the cross-neutralizing capacity of IVIg batches composed of plasma from the general population of the Netherlands during 2005–2014 against EV-71 subtypes circulating in Europe or Asia and compared results to IVIg batches from Japanese and Vietnamese donors. Furthermore, we determined neutralizing antibody (nAb) titers against EV-71 in serum samples from Dutch donors representing groups that are vulnerable for EV-71 infection but that are not or might not be present in large numbers in the IVIg pools. These include children <5 years of age, the main target group for enterovirus infections, and women of childbearing age who had a high probability of exposure by contact with young children. We also included serum samples from HIV-infected men in the analyses to determine EV-71 seroprevalence in a background population with an estimated average exposure to enteroviruses and without increased risk for enterovirus infections. Analysis of the complete capsid encoding regions of EV-71 strains included in the serologic analyses provided possible explanations for observed differences in nAb titers.

## Materials and Methods

### Cells and Virus Strains

Serum neutralization assays were performed by using rhabdomyosarma (RD), human colorectal adenocarcinoma (HT-29) and African green monkey kidney (Vero) cells (American Type Culture Collection, https://www.atcc.org/). Cell lines were cultured at 37°C, 5% carbon dioxide (CO_2_) in Eagle’s minimum essential medium (EMEM; Lonza, Verviers, Belgium) supplemented with 10% fetal bovine serum (FBS, Sigma-Aldrich, Zwijndrecht, Netherlands), 100 IU/mL of penicillin, and 100 μg/mL of streptomycin. EV-71 strains C1 91–480 and C2 07–2485 (provided by the National Institute for Public Health and the Environment, Bilthoven, The Netherlands), were isolated from clinical specimens in 1991 and 2007, respectively, by the national enterovirus surveillance system in the Netherlands (*10*,*18*). Strains C2 2105–1721 and C2 2105–2503 were isolated as part of primary diagnostics at the Academic Medical Center (AMC) in Amsterdam in 2010. The B3 SK-EV006 and B4 C7-Osaka strains were isolated in Malaysia and Japan in 1997. Strain C4 75-Yamagata was isolated in Japan in 2003, and strain C5 209-VN in Vietnam in 2006.

### IVIg Batches and Serum Samples

IVIg batches composed of plasma from Dutch or Asian donors were tested for their cross-neutralizing capacity against EV-71 strains isolated in the Netherlands and Asia. Six batches from Dutch donors were included (Nanogam, Sanquin, the Netherlands): 1 each from 2005, 2009, and 2014 and 3 from 2010. Batches from Asian donors were 2 from Japan (Teijin Institute for Bio-Medical Research, Hino, Tokyo, Japan; year of manufacture unknown) and 1 from Vietnam from 2011 (Green Cross Corporation, Pymepharco, Vietnam). Along with the IVIg batches, a previously described rabbit polyclonal serum against EV-71 C1 91–480 was included in the neutralization assays (*16*). Individual human serum samples used for this study were collected and stored at −20°C as part of primary virus diagnostics in the Laboratory of Clinical Virology at AMC during 2010–2014. We defined 2 groups vulnerable for EV-71 infection: children <5 years of age, and women of childbearing age who were admitted to the obstetrics ward and had a high probability of exposure to young children. HIV-positive men receiving treatment who regularly attended the outpatient clinic for HIV care were included to study seroprevalence in a background population. A total of 177 samples (on average 12 serum samples/year) were randomly selected from these groups; those from children <5 years of age showed a proportional distribution of ages for each year. None of the patients selected had been diagnosed with hand, foot and mouth disease or had positive results for routine EV diagnostic tests. 

According to laws in the Netherlands, no ethical approval is required for anonymous use of biobanked specimens. The study was conducted according to the Dutch code of conduct for responsible use of human tissue for medical research 2011 (http://www.federa.org/code-goed-gebruik-van-lichaamsmateriaal-2011) and the AMC Research Code (https://www.amc.nl/web/AMC-website/Research-Code/).

### Serum Neutralization Assays

EV-71 nAb titers of IVIg batches and individual human serum samples were determined by using a serum neutralization assay. Human serum samples were heat inactivated at 56°C for 30 min. A 2-fold serial dilution of the serum samples was subsequently incubated with an equal volume of chloroform-treated 100 50% cell culture infectious doses of virus at 37°C in 5% CO_2_ for 1 h. HT29, RD, or Vero cells in EMEM supplemented with 10% FBS were subsequently added and plates were incubated at 37°C in 5% CO_2_ for 5 d. The neutralizing titer was calculated on the basis of the number of wells showing cytopathogenic effect by using the Spearman-Karber method and reported as the reciprocal titers of serum dilutions that exhibited 50% neutralization. A neutralizing titer of >1:16 was used as a threshold for seropositivity, because this titer has been correlated with protection against EV-71–associated disease in phase III clinical trials with EV-71 vaccines (*2*).

### Plaque Assay

Preliminary data indicated that several EV-71 strains escaped neutralization by IVIg batches and individual human serum samples. To exclude presence of non–EV-71 virus strains in our virus stocks, 3,000 50% cell culture infectious doses of the EV-71 virus strains escaping neutralization and a strain that was neutralized as a positive control was incubated with a 1:4 dilution of the rabbit polyclonal serum against EV-71 C1 91–480, a selection of the IVIg batches, or plain medium (control) at 37°C in 5% CO_2_ for 1 h. In total, 200 μL of a 0.5 log_10_ serial dilution of the virus and serum mixtures was transferred to a monolayer of Vero cells in a 6-well format and incubated at 37°C in 5% CO_2_ for 1 h. Cells were subsequently covered with 0.9% agarose mixed with 2× concentrated MEM supplemented with 4% fetal calf serum (1:1 ratio). Plates were incubated at 37°C at 5% CO_2_ for 48–72 h. Single plaques were harvested and amplified on Vero cells before RNA isolation by using the GenElute Mammalian Total RNA Miniprep Kit (Sigma-Aldrich, Zwijndrecht, the Netherlands) according to the manufacturer’s instructions. The viral RNA was subsequently subjected to an enterovirus genotyping PCR as described previously (*19*).

### Capsid Sequence Analysis

Viral RNA was extracted from 50 μL of EV-71 isolates cultured on RD, Vero, or HT-29 cell lines, by using the GenElute Mammalian Total RNA Miniprep Kit (Sigma-Aldrich), according to the manufacturer’s instructions. Viral RNA was eluted in 50 μL elution buffer. The capsid-encoding regions were PCR amplified in 4 overlapping regions by using genogroup B- and C-specific primers and a PCR amplification protocol described previously (*20*). Sequencing of the PCR products was performed by using the ABI Prism BigDye Terminator Cycle Sequencing Ready Reaction Kit version 3.2 (Applied Biosystems, Foster City, CA, USA) on an automated sequencer (Applied Biosystems). Editing of the sequence data and generation of consensus sequences of the capsid region by assembling overlapping DNA sequences determined from both strands was performed by using the ClustalW method implemented in BioEdit version 7.2.5 (*21*). Nucleotide sequences of EV-71 strains used in the current study are accessible in the Genbank/EMBL/DDBJ nucleotide sequence databases under accession numbers AB552982.1 (C1 91–480), AB552987.1 (C2 2485), KU697333 (C2 1721), KU697334 (C2 2503), KU697335 (C4 75-Yamagata), KU697336 (C5 209-VN), AB469182.1 (B3 SK-EV006), and AB550336.1 (B4 C7-Osaka).

### Statistical Analysis

Differences in titers among the study groups were analyzed by using the Kruskal-Wallis One-Way ANOVA test in GraphPad Prism 5 software (GraphPad Software, San Diego, CA, USA) at a significance level of p<0.05. The 95% CIs of the proportions of seropositive persons were calculated according to the E.B. Wilson method, using the VassarStats Web site for Statistical Computation (http://vassarstats.net/index.html).

## Results

### EV-71 Cross-neutralizing Capacity of IVIg Batches

**Figure 1:**
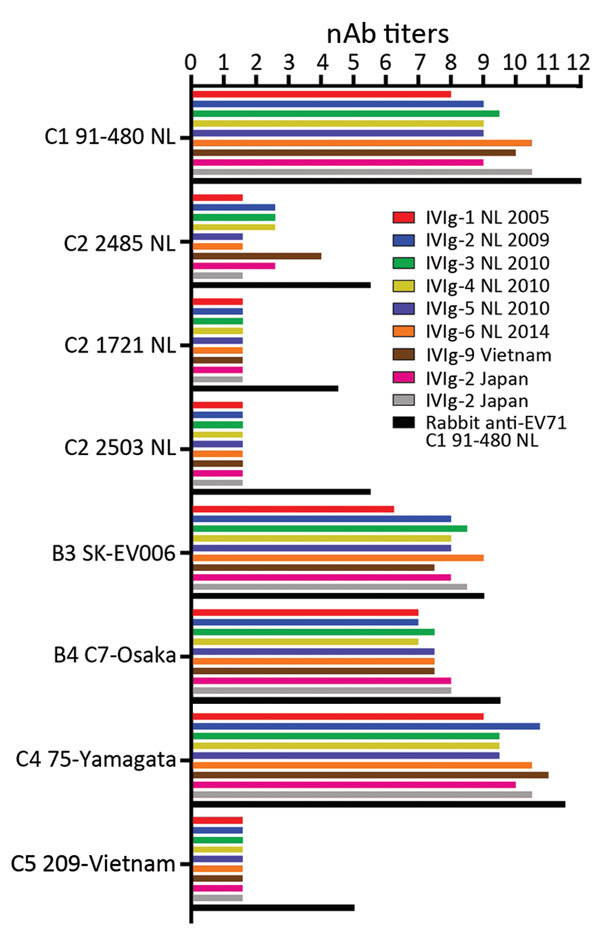
EV-71 nAb titers in IVIg batches composed of plasma from Dutch (6 batches), Japanese (2 batches), and Vietnamese (1 batch) donors and in a polyclonal rabbit serum against EV-71 C1 91–480. nAb titers are presented as log_2_ values. EV-71, enterovirus 71; IVIg, intravenous immunoglobulin; nAb, neutralizing antibody; NL, the Netherlands.

As human immunoglobulin batches represent the immunological profile of the general population, we studied the cross-neutralizing capacity of IVIg batches composed of plasma from Dutch, Japanese, and Vietnamese donors against EV-71 strains isolated in Europe and Asia (Figure 1). All batches had high nAb titers against the Dutch C1 strain (mean titer 1:776) and the Asian C4, B3, and B4 strains (mean titers 1:1176, 1:274, and 1:181, respectively). However, no neutralizing efficacy was observed against the Dutch C2 strains (isolated in 2007 and 2010) and the Asian C5 strain in any of the batches, including the Vietnamese IVIg batch. The hyperimmune rabbit polyclonal serum against EV-71 C1 91–480 did neutralize the strains, but with several-fold lower titers than observed for C1, C4, B3, and B4 strains (Figure 1). The results remained similar when virus strains were pretreated with chloroform to remove potential virus aggregates or when a different cell line (Vero) was used (data not shown). To exclude presence of non–EV-71 strains in our virus stocks that caused the cytopathogenic effect in the neutralization assays, we plaque-purified viruses incubated with or without the polyclonal serum from rabbits and a selection of the IVIg batches. Genetic characterization confirmed the presence of the EV-71 C2 and C5 genotypes in the virus stocks that escaped neutralization.

### EV-71 Neutralizing Antibody Titers in Serum from Dutch Donors

**Figure 2:**
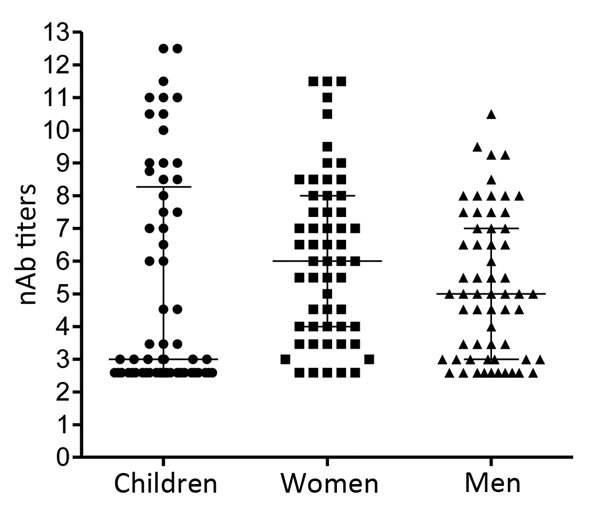
Enterovirus 71 nAb titers in serum collected from Dutch children ≤5 years of age, women of childbearing age, and HIV-positive men during 2010–2014. nAb titers are presented as log_2_ values. Median titers (wide horizontal lines) with interquartile ranges (error bars) are indicated for each category. nAb, neutralizing antibody.

**Figure 3:**
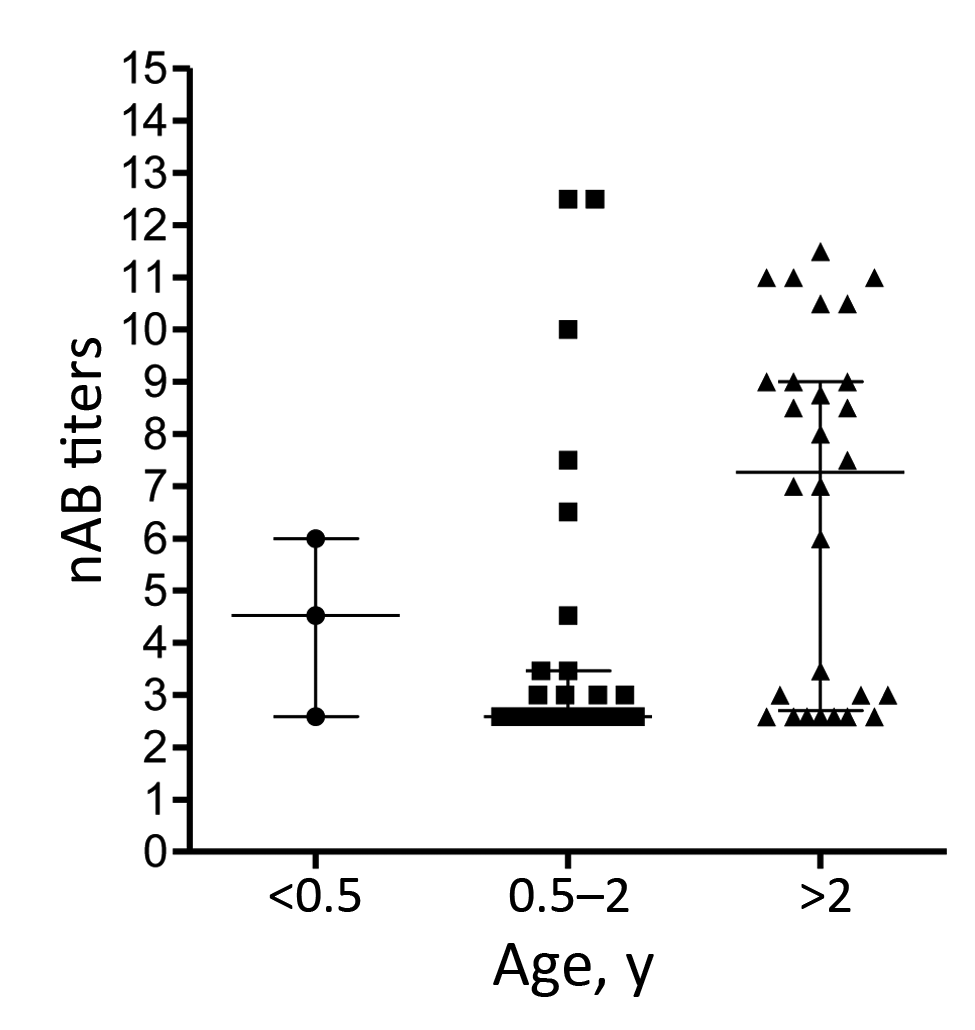
Enterovirus 71 nAb titers in serum collected from Dutch children (<0.5 years, 0.5–2 years and >2–5 years of age) during 2010–2014. nAb titers are presented as log_2_ values. Median titers (wide horizontal lines) with interquartile ranges (error bars) are indicated for each category. nAb, neutralizing antibody.

To determine the level of protection in groups at risk for EV-71 infection, nAb titers against the Dutch C1 strain were determined in serum samples from 61 children (median age 2 years, interquartile range [IQR] 1–4 years) and 56 women of child-bearing age (median age 28.6 years, IQR 24.4–34.3 years), collected in the Amsterdam area during 2010–2014. Additionally, serum samples from 60 HIV-positive men (median age 38.2 years, IQR 30–51.7 years), collected during the same years in this region, were included to study seroprevalence in a background population. On average, 41% (25/61) of the children had nAb titers (>1:16) against EV-71 C1 (Table 1; Figure 2). Of the 61 children, 3 were <0.5 years of age; 2 of those had nAb titers against EV-71, most likely reflecting presence of maternal antibodies. The remaining 58 children were ≥0.5–5 years of age; the percentage of seropositive children was highest in the age group >2–5 years (60.7% vs. 20% in the age category 0.5–2 years). The percentages of seropositive children varied per year. In 2011, 4 (57%) of the 7 children 0.5–2 years of age who were tested were found to be seropositive, compared to none in 2010 and 2014. The nAb titers among children 0.5–2 years of age (median titer 1:6) were significantly lower than those for children >2–5 years of age (median titer 155; p<0.05) (Figure 3). 

**Table 1:** Percentages of children <5 y of age, women of childbearing age, and HIV-positive men with nAb titers against the Dutch enterovirus 71 strain C1 91–480, the Netherlands*

Year	No. positive/no. tested (%, 95% CI)						
	Children, age, y					Adults, characteristics	
	<0.5	0.5–2	>2–5	Total ≤5		F, childbearing age	M, HIV-positive
2010	1/1 (100, 20.6–100)	0/7 (0, 0–35.4)	3/5 (60.0, 23.1–88.2)	4/13 (30.8, 12.7–57.6)		10/13 (76.9, 49.7–91.8)	9/14 (64.3, 38.8–83.7)
2011	1/2 (50.0, 9.5–90.6)	4/7 (57.1, 25.0–84.2)	3/6 (50.0, 18.8–81.2)	8/15 (53.3, 30.1–75.2)		8/10 (80.0, 49.0–94.3)	5/10 (50.0, 23.7–76.3)
2012	0/0	1/8 (12.5, 2.2–47.1)	5/6 (83.3, 43.7–97.0)	6/14 (42.9, 21.4–67.4)		9/12 (75.0, 46.8–91.1)	10/14 (71.4, 45.4–88.3)
2013	0/0	1/3 (33.3, 6.2–79.2)	4/8 (50.0, 21.5–78.5)	5/11 (45.5, 21.3–71.2)		6/8 (75.0, 40.9–92.9)	10/14 (71.4, 45.4–88.3)
2014	0/0	0/5 (0, 0.0–43.5)	2/3 (66.7, 20.8–93.9)	2/8 (25.0, 7.2–59.1)		11/13 (84.6, 57.8–95.7)	5/8 (62.5, 30.6–86.3)
Total	2/3 (66.7, 20.8–93.9)	6/30 (20.0, 9.5–37.3)	17/28 (60.7, 42.4–76.4)	25/61 (41.0, 29.5–53.5)		44/56 (78.6, 66.2–87.3)	39/60 (65.0, 52.4–75.8)
*nAb, neutralizing antibody.							

Among women of childbearing age, 44 (79%) of 56 were seropositive (median titer 1:64) (Figure 2). Titers within this category significantly differed from those of children 0.5–2 years of age (p<0.05) but not from titers of children >2–5 years of age. 

Of 60 HIV-positive men, 39 (65%) were seropositive for EV-71 (median titer 1:32). Although titers were lower within this group, they did not significantly differ from those observed among children >2–5 years of age or among women of childbearing age.

### Cross-neutralizing Capacity of Human Serum

A selection of individual serum samples with nAb titers against the EV-71 C1 strain were tested for cross-neutralizing activity against heterotypic EV-71 genotypes (Table 2). A total of 17 seropositive serum samples (from 7 children, 4 women, and 6 men) with nAb titers ranging from 1:91 to 1:2,896 (mean 1:997), were selected for inclusion on the basis of the volume available for testing multiple EV-71 strains and the titer being high enough to detect several folds lower heterologous nAb titers. Four seronegative serum samples with nAb titers of <1:8 from 2 children and 2 men were included to study whether serum samples with no nAbs against EV-71 C1 potentially contained nAbs against other EV-71 types. In line with nAb titers of the IVIg batches, all serum samples from the 17 EV-71 seropositive donors showed nAb titers against the C4 strain in the range of those observed for the C1 strain (maximum 2-fold differences, mean titer 1:1,024). High nAb titers, but several-fold lower than those for C4 strains, were observed for B3 and B4 strains (mean 1:239 and 1:158, respectively). However, the Dutch C2 1721 strain and Vietnamese C5 strain could not be neutralized by any of the serum samples. Serum samples of the 4 donors with nAb titers of <1:8 against the Dutch C1 strain did not neutralize any of the strains tested.

**Table 2:** Cross-neutralization of heterotypic Asian and Dutch enterovirus 71 strains by serum from Dutch donors, the Netherlands*

Serum sample no.	Category	nAb titers against					
		C1 91–480 NL	C2 1721 NL	C4 75-Yamagata	C5 209-VN	B3 SK-EV006	B4 C7-Osaka
1	Children	1,448	NT	1,448	NT	362	64
2	Children	362	NT	362	NT	128	45
3	Children	2,048	NT	1,448	NT	724	362
4	Children	1,448	NT	1,024	NT	512	256
5	Children	2,896	NT	2,048	NT	ND	ND
6	Children	2,048	NT	5,793	NT	ND	ND
7	Children	181	NT	ND	ND	ND	ND
8	Women	2,896	NT	1,448	NT	181	256
9	Women	91	NT	91	NT	45	23
10	Women	724	NT	724	NT	128	362
11	Women	181	NT	256	NT	32	32
12	Men	724	NT	512	NT	181	64
13	Men	609	NT	362	NT	64	91
14	Men	609	NT	512	NT	ND	ND
15	Men	256	NT	181	NT	ND	ND
16	Men	256	NT	181	ND	ND	ND
17	Men	181	NT	128	NT	ND	ND
18	Men	8	NT	NT	NT	ND	ND
19	Men	8	NT	NT	NT	ND	ND
20	Children	8	NT	NT	ND	ND	ND
21	Children	8	NT	NT	NT	ND	ND
*ND, not determined; NT, no titer.							

### Amino Acid Sequence Comparison

**Figure 4:**
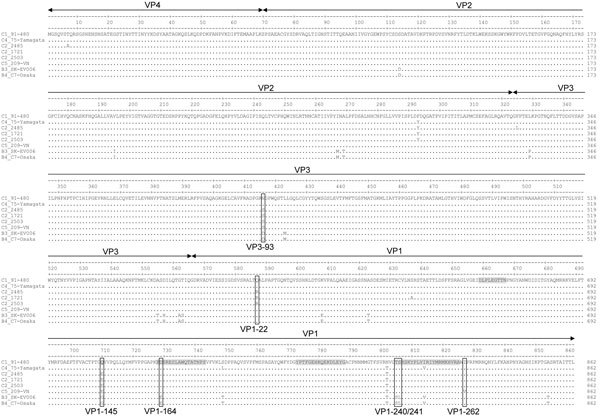
Amino acid sequence comparison of the capsid regions of EV-71 strains used for serologic analysis of strains isolated in Asia and Europe in study of immunity to these strains after exposure to IVIg from viruses from the Netherlands. Residues 164, 240, and 241 of VP1 (differing among genogroup B and C viruses and located in known antigenic sites) and residues that differ among neutralizable and nonneutralizable strains are marked by boxes. Known epitopes and antigenic determinants for EV-71 are marked in gray (22–25). EV-71, enterovirus 71; IVIg, intravenous immunoglobulin; VN, Vietnam; VP, viral protein.

To find explanations for the escape of C2 and C5 strains from neutralization, the complete capsid encoding regions of strains included in this study were sequenced and used for amino acid sequence analysis (Figure 4) (*22*–*25*). In total, 19 residues differed between the genogroup B and C strains, of which 3 were located in a linear epitope: residues 164, 240, and 241 of VP1 (Figure 4) (*22*–*25*). The C2 and C5 strains differed from strains that could be neutralized in residues 145 of VP1 (145E vs. 145G/Q) and 93 of VP3 (93S vs. 93N/D). The C2 strains had an additional mutation of residue 22 of VP1 (22R vs. 22Q) and the C5 strain of residue 262 of VP1 (262V vs. 262I).

## Discussion

We determined the nAb titers against EV-71 in IVIg batches and individual serum samples from Dutch donors and tested their cross-neutralizing capacity against strains responsible for outbreaks in Asia. The high percentage of EV-71 seropositive serum samples suggests widespread circulation of EV-71 in the Netherlands, which is likely sustained by the presence of a relatively large cohort of susceptible infants (80% in the age category 0.5–2 years). In 2010, EV-71 caused an elevated number of enterovirus infections in the Netherlands, likely explaining the high number of seropositive children in 2011 (*18*) and in the age category >2–5 years in 2012 in this study.

The rates of seropositivity observed in this study are comparable to those observed among the German population (*26*,*27*). The prevalence, however, seems not be uniform across Europe, because seroprevalence levels among children in Finland (<11 years of age) and in European regions of Russia (3–5 years of age) were only 1.6% and 19%–27%, respectively (*6*,*28*). The seroprevalence levels observed in the Netherlands are in the range of those observed in Asia before outbreaks (*29**–**33*). Although this finding points toward the possibility of large outbreaks in Europe as well, widespread circulation of Asian EV-71 genotypes and associated outbreaks seem to be restricted to the Asian region. 

In this study, we showed that IVIg batches and serum samples with nAb titers against the Dutch C1 strain efficiently neutralized Asian C4, B3, and B4 strains. This result implies that administration of IVIg from the Netherlands could benefit recovery of a patient infected by an Asian EV-71 strain and could explain why the multiple introductions of genotype C4 strains in Europe have not resulted in widespread circulation (*6*,*9*,*10*). In agreement with this finding, the transient occurrence of C4 and B5 infections in Europe coincided with low population sizes of C1 and C2 (*34*). However, a relatively large group of susceptible children is still at risk for severe EV-71 infections; therefore, monitoring introduction of new genotypes into Europe remains of importance.

A limitation of our study is that it is not possible to differentiate between neutralization and cross-neutralization. The high neutralizing activity against C4 strains could reflect actual exposure to C4 virus rather than cross-neutralization of antibodies induced by C1 or C2 strains. This possibility, however, is thought to be negligible, because the systematic, nationwide enterovirus surveillance system in the Netherlands has only reported circulation of C1 and C2 strains (*10*). Additionally, we showed neutralization of the C4 strain by a polyclonal rabbit serum against EV-71 C1, which confirms that nAbs elicited against C1 are cross-protective against C4. We did not have access to antigenically divergent genotype B5 strains, which is unfortunate, as B5 strains were isolated from clinical specimens in Denmark in 2007 and in France in 2013 (*8*).

Neither the C2 strains nor the C5 strain could be neutralized by IVIg batches or individual serum samples. Because C2 has been persistently circulating in Europe since 1997 (*10*), it is unlikely that there has not been exposure to this genotype. In fact, a serum sample from our biobank obtained from a patient proven to be infected with EV-71 C2 could not neutralize the Dutch C2 strains either (data not shown). Large variations in nAb titers against C2, even in serum from children proven to be infected with C2, were observed by other research groups as well (*11*,*12*,*35*,*36*). Variation in neutralizing activity could be explained by antigenic diversity among strains included in the analyses and strains to which patients or populations were exposed (*23*,*37*). The C2 and C5 strains escaping neutralization in the current study differed from the neutralizable strains in VP3 residue 93 and VP1 residues 22 and 145 (C2) or 145 and 262 (C5). VP1 residue 145 has previously been identified as a key antigenic determinant, of which substitution can significantly affect the neutralizing activity (*23*,*38*). Furthermore, VP1 22, 145, and 262 are among residues of which substitution through time has been suggested to be necessary for EV-71 persistence by generating antigenic novelty (*5*). Because the evolution of EV-71 genotypes is shaped by a continuous replacement of viral lineages over time, it will be of importance to further characterize the role of the identified mutations in determining antigenic diversity and to study whether the observed low nAbs titers against C2 and C5 reflect a real low seroprevalence or are a test artifact due to inclusion of antigenically divergent strains. 

Cross-neutralizing activity against C5 in serum of C4-infected humans has been reported, and from that perspective it is remarkable that the C5 strain escaped neutralization by the IVIg isolated in Vietnam that had high nAb titers against C4 (*12*,*39*,*40*). However, it is complicated to compare results from studies that used different virus strains and serum from persons with different exposure histories, considering the co-circulation of different genotypes and the continuous evolution of EV-71 types with potentially novel antigenicity (*5*).

In conclusion, we showed the presence of high cross-nAb titers against EV-71 in IVIg batches and serum samples from Dutch donors. This finding implies that administration of Dutch IVIg could support recovery of a patient infected with an Asian EV-71 strain and that herd immunity induced by locally circulating strains could be cross-protective against widespread circulation and associated outbreaks of Asian strains in Europe. The identification of viruses that escape neutralization, however, warrants further research on antigenic diversity among EV-71 strains and emphasizes the importance of monitoring both the genetic and antigenic diversity of circulating strains.
